# miR-375 activates p21 and suppresses telomerase activity by coordinately regulating HPV E6/E7, E6AP, CIP2A, and 14-3-3ζ

**DOI:** 10.1186/1476-4598-13-80

**Published:** 2014-04-08

**Authors:** Hyun Min Jung, Brittany L Phillips, Edward KL Chan

**Affiliations:** 1 Department of Oral Biology, University of Florida, 1395 Center Drive, Gainesville, FL 32610, USA

**Keywords:** MicroRNA, miR-375, p21, Human papillomavirus, E6AP, Telomerase

## Abstract

**Background:**

While microRNAs (miRNAs) are extensively studied in post-transcriptional regulation of gene expressions in many biological processes, cellular miRNA-mediated regulation of viral genes remains unclear. In particular, the interplay between human papillomavirus (HPV) genes and miRNAs and how these interactions contribute to HPV-associated cancers remain elusive.

**Methods:**

Transient transfection of miR-375-mimic was used to compensate the loss-of-function of miR-375 in HPV-positive cancer. Regulation of oncogenic molecules and their downstream molecules via miR-375 in HPV-positive cancer was investigated using qRT-PCR, western blot, dual luciferase assay, indirect immunofluorescence analysis. All experiments were conducted at least three times to achieve statistical significance determined by Student t-test.

**Results:**

In this study, we demonstrated how miR-375 negatively regulates HPV16 and 18 transcripts. We also found a cellular protein, E6-associated protein (E6AP), directly regulated by miR-375. miR-375-mediated repression of HPV transcripts and E6AP elevated major tumor suppressors p53, p21, and retinoblastoma protein 1 (RB). Cooperative regulation of miR-375 targets along with the increase of tumor suppressors led to ~60% reduction of telomerase reverse transcriptase (TERT) transcription followed by ~35% decrease of telomerase activity. Furthermore, miR-375-mediated regulation of 14-3-3ζ contributes to decrease telomerase activity by altering nuclear translocation of TERT.

**Conclusion:**

Taken together, miR-375-mediated suppression of multiple oncogenic components in HPV-associated carcinogenesis generates a cumulative biological response to rescue key tumor suppressors and diminish telomerase activity, which results in cell cycle arrest and cell proliferation inhibition.

## Introduction

MicroRNAs (miRNAs) are approximately 22 nucleotide (nt) long small non-coding RNAs that play key roles in differentiation and development by post-transcriptional regulation of cellular genes [1]. Although typical miRNA-mRNA interactions occur in the 3′ untranslated region (UTR) of the target, coding sequences are also subjected to miRNA binding. This binding directs translational repression and mRNA degradation [2,3]. Over 1,000 human miRNAs are predicted to regulate approximately 60% of protein-coding genes, indicating their extensive functions in many biological processes [4].

Human papillomaviruses (HPVs) are 8-kb double-stranded DNA viruses that specifically target basal cells of the epithelial mucosa [5]. HPV infection is implicated in virtually all cervical cancers, ~90% of anal cancers, 30 ~ 60% of oropharyngeal cancers, and ~40% of vaginal, vulvar, and penile cancers [6]. HPV16 and HPV18 are the most common HPV types associated with these aforementioned cancers [7]. High-risk HPVs closely linked to malignancies have developed strategies to evade the human immune system. Examples include suppressing interferon-α-inducible gene expression [8,9], and directly regulating interferon signaling pathways by HPV E6/E7 [10,11]. While little is known about the role of E1 in HPV-mediated cancers, E6 and E7 coded by high-risk HPVs are considered highly oncogenic [5,7,12-15].

Telomeres are specialized functional complexes that maintain the integrity and stability of the genome by protecting the ends of eukaryotic chromosomes [16]. A telomere consists of hexanucleotide (TTAGGG) tandem repeats, ending with a single stranded overhang that forms telosomes with protein complexes [17]. In normally dividing somatic cells, telomeres shorten by about 60-150 bp with each cell division due to the DNA end replication problem [18,19]. Then telomeres are shortened to a certain length, cell cycle arrest and proliferation failure occur, inducing cellular senescence [20]. Tumors can overcome this regulation by activating telomerase, a ribonucleoprotein complex enzyme with reverse transcriptase activity that maintains a constant telomere length [21,22]. Enhanced telomerase activity is observed in most malignant tumors (~90%) but the mechanisms underlying its regulation are not fully understood [23].

In the present study, we showed that miR-375 suppresses high-risk HPV E6 and E7, and E6AP. Moreover, the effect of these regulations was incorporated with previous findings of miR-375 targets shown in other cancer cells [3,24]. This study demonstrates how miR-375 behaves as a tumor suppressor miRNA by rescuing major tumor suppressors, suppressing oncogenic components, and thus regulates cell cycle and telomerase activity.

## Results

### miR-375 targets HPV type 16 and 18 transcripts and suppresses E6 and E7 expression

**Figure 1:**
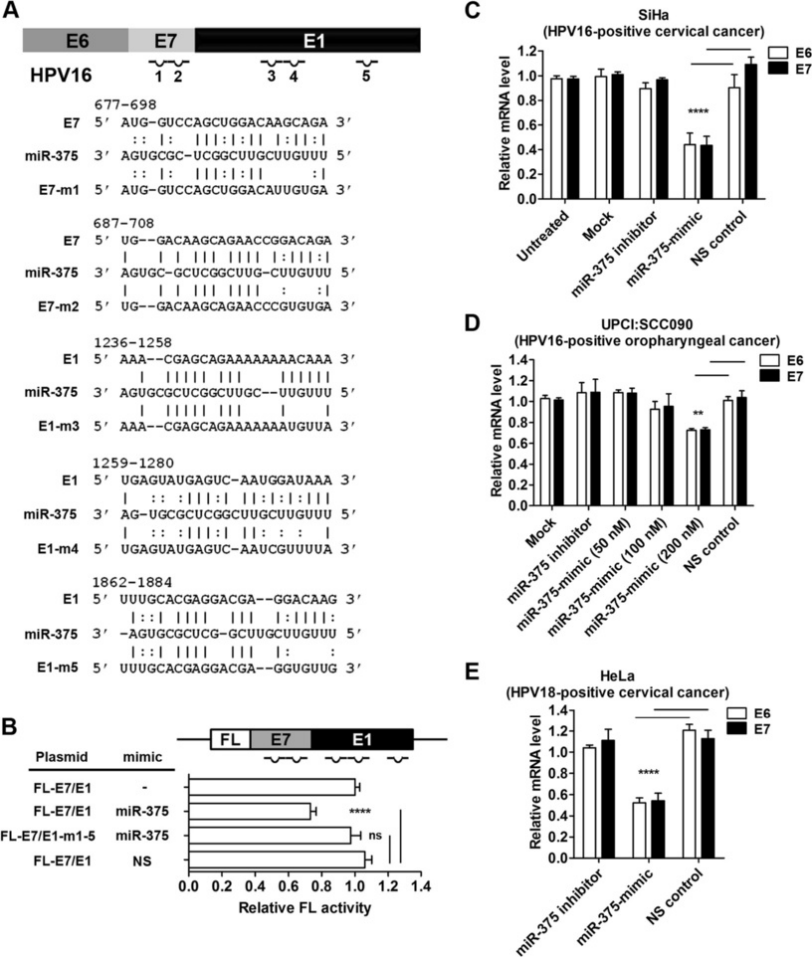
**miR-375 targets HPV type 16 and 18 transcripts and suppresses E6 and E7 expression. (A)** Five putative miR-375 binding sites on HPV16 transcript sequence. Integrated HPV16 viral gene produces polycistronic transcripts consisting of E6, E7, and E1. Two putative sites on E7 region and three putative sites on E1 region of HPV16 transcript were predicted to interact with miR-375. Lines between nucleotides of HPV16 sequence and miR-375 are typical Watson–Crick interactions (A-U and G-C, respectively), and colons are weak, nontypical base-pair interactions. Corresponding specific seed binding site mutants were generated for dual luciferase reporter assays. **(B)** FL repression effect was analyzed with FL construct that contains both E7 and E1 regions. Five putative miR-375 binding sites were mutated to examine their direct interaction. Cells were cotransfected with miR-375-mimic or non-specific control (NS). *Renilla* luciferase was used as an internal control to normalize FL activity expression. Results were compared to NS control transfected cells for statistical analyses. **(C)** Real-time PCR analyses of endogenous E6 and E7 mRNA levels in SiHa (HPV16-positive cervical cancer cell line) transfected with miR-375-mimic. **(D)** Measurement of endogenous E6 and E7 mRNA level in UPCI:SCC090 (HPV16-positive oropharyngeal cancer cell line) transfected with miR-375-mimic. Final concentration of 25 nM was used for miR-375 inhibitor and NS control, and 50, 100, 200 nM were used for miR-375-mimic transfection. **(E)** Expression changes in E6 and E7 mRNA in HeLa (HPV18-positive cervical cancer) after miR-375-mimic transfection. Final concentration of 25 nM was used for miR-375 inhibitor, miR-375-mimic, and NS control for transfection. White and black bars indicate E6 and E7 mRNA levels, respectively. Results are expressed as mean ± SD from at least three independent experiments. **p < 0.01 and ****p < 0.0001.

While searching for oncogenic targets regulated by miR-375 in addition to cancerous Inhibitor of PP2A (CIP2A) in our previous study [3], we investigated potential interactions between miR-375 and HPV16 transcripts. We used the program RNA22 to predict these potential interactions because this algorithm relies on sequence complementarity between miRNA and target RNA regardless of interaction location within the transcript [25]. A total of five putative miR-375 binding sites were predicted, two in the E7 region and three in the E1 region (Figure 1A). We generated FL reporter constructs to determine the direct interactions between miR-375 and these predicted sites. We inserted E7/E1 region downstream of the FL reporter (Figure 1B). Site-directed mutagenesis generated mutations in the seed sequences of five miR-375 binding sites (Figure 1A and 1B). Co-transfection of FL-E7/E1 reporter with miR-375-mimic showed 25-30% repression, and the repression effect was abolished when the five miRNA-mRNA interactions were disrupted (FL-E7/E1-m1-5, Figure 1B). Co-transfection of FL-E7 with miR-375-mimic resulted in ~20% reduced FL activity, but mutation of individual binding sites (m1 or m2) did not rescue this repression effect (Additional file 1: Figure S1A). Similar results were observed when FL-E1 was co-transfected with miR-375-mimic. FL activity was significantly reduced (~20%) by miR-375 but mutations on single miR-375 binding sites (m3 to m5) did not rescue this repression (Additional file 1: Figure S1B). These results indicate that the five binding sites more likely produce a synergistic effect, instead of an additive effect, to suppress HPV16 transcript expression.

In order to measure the repression effect of miR-375 on endogenous HPV16 transcripts, two HPV16-positive cancer cell lines, cervical cancer line SiHa and oropharyngeal cancer line UPCI:SCC090 (hereafter SCC090), were transfected with miR-375-mimic. E6 and E7 mRNA expression were measured 48 h post-transfection using Taqman quantitative PCR. Interestingly, both E6 and E7 transcript levels were reduced ~60% in SiHa cells transfected with 25 nM of miR-375-mimic molecules (Figure 1C). In SCC090 cells, neither 50 nor 100 nM of miR-375-mimic was sufficient to reduce E6 or E7 levels. Significant reduction of both E6 and E7 (~30%) was observed when a higher concentration (200 nM) of miR-375-mimic was used for transfection (Figure 1D). This higher dose requirement in SCC090 cells could be explained by the dramatically higher HPV16 transcript levels present compared to SiHa cells (Additional file 1: Figure S2). E6, E7, and E6/E7/E1 transcripts were detected in both cell lines, as well as in three human oropharyngeal tumors (T1-T3) (Additional file 1: Figure S2). Although four E6 mRNA splicing isoforms were detected as reported previously [26], the miR-375-mediated repression observed here is not affected since the putative miR-375 binding sites are located on E7 and E1 region. Interestingly, miR-375-mimic transfection in HeLa cells (HPV18 positive) also reduced both E6 and E7 transcript levels by ~50% (Figure 1E). Bioinformatics suggested that HPV18 also have putative binding sites for miR-375 (data not shown). Transfection of miR-375 inhibitor did not affect the expression of HPV16 and HPV18 transcript expressions in SiHa, SCC090, and HeLa cells because endogenous miR-375 levels in these cells were already extremely low (~32 Ct values by real-time PCR). However, the functionality of the miR-375 inhibitor was clearly demonstrated in the transfection of MCF7 cells which had moderate expression of miR-375 (see in the following figures). Taken together, these data indicate that miR-375 directly regulates HPV16 and HPV18 transcripts.

### miR-375 directly targets and represses E6AP

**Figure 2:**
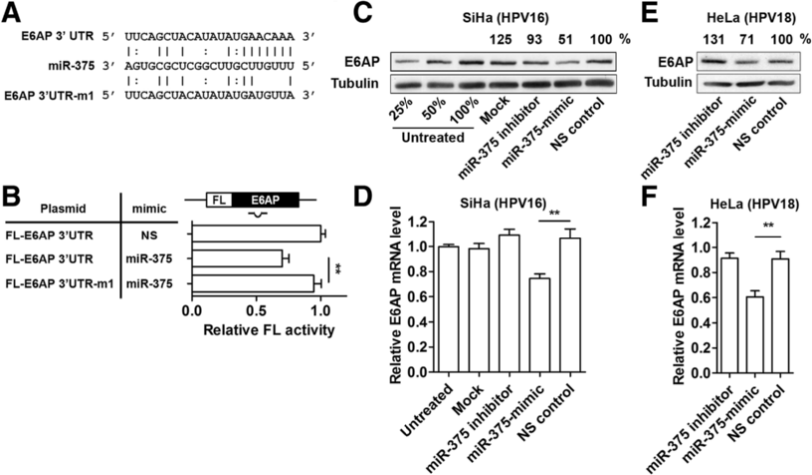
**miR-375 directly targets and represses E6AP. (A)** Sequence of putative miR-375 binding site in the 3′UTR of E6AP. E6AP 3′UTR-mut was generated by disrupting base paring of four nucleotides, positions 2 to 5. **(B)** Relative FL repression effects of miR-375 on the FL-E6AP reporter. Results were compared with FL-E6AP co-transfected with NS control for statistical analysis. **(C)** E6AP protein level changes in SiHa cells transfected with miR-375 inhibitor, -mimic, or NS control were analyzed by Western blot. Tubulin expression was used as internal control. 25%, 50%, 100% amounts of untreated cell lysates were included to calibrate the semiquantitative analysis. **(D)** In parallel, relative endogenous mRNA levels of E6AP from SiHa cells transfected with miR-375 inhibitor, -mimic, or NS control were measured using qRT-PCR. **(E)** E6AP protein level in HeLa cells transfected with miR-375 inhibitor, -mimic, or NS control was analyzed by Western blot. **(F)** Endogenous E6AP mRNA level in HeLa cells were measured 48 h post-transfection. Results are expressed as mean ± SD from at least three independent experiments. **p < 0.01.

Prediction of miR-375 binding site in the E6AP 3′UTR was performed by TargetScan [27]. The 3′UTR of E6AP was cloned downstream of FL reporter and site-directed mutagenesis of four nucleotides in the seed binding sites was also generated to test the putative miRNA-mRNA interaction (Figure 2A). The dual luciferase reporter system was used to investigate the direct interaction between E6AP 3′UTR and miR-375. A significant decrease in FL activity (30%, p <0.01) was observed. This direct repression effect was abolished by mutating the seed binding site (Figure 2B). SiHa cells were used in the following experiments as HPV16-positive cells due to the lower concentration of miR-375-mimic required for HPV16 suppression in SiHa compared to SCC090 cells (Figure 1). Transfection of miR-375-mimic (25 nM) in SiHa cells reduced ~50% of E6AP protein levels (Figure 2C) and ~30% of endogenous E6AP mRNA (Figure 2D). Transfection of miR-375-mimic (25 nM) in HeLa cells reduced ~30% of E6AP protein and ~40% of E6AP mRNA (Figure 2E and 2F). These results demonstrate that miR-375 directly regulates E6AP by interacting with the 3′UTR.

### miR-375 increases p21, p53, and RB in HPV16- and 18-positive cancer

**Figure 3:**
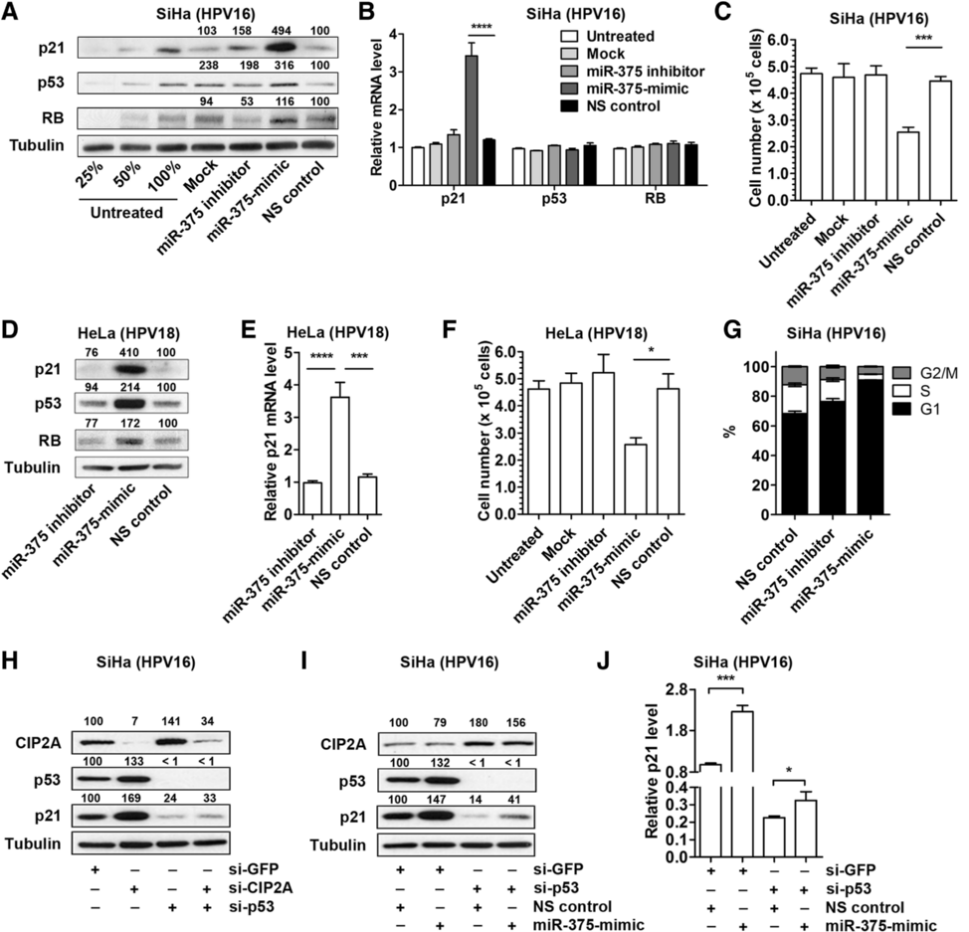
**miR-375 increases p21, p53, and RB in HPV16- and 18-positive cancer. (A)** Protein levels of p21, p53, and RB in SiHa cells transfected with miR-375 inhibitor, -mimic, or NS control were measured by Western blot analysis. 25%, 50%, 100% amounts of untreated cell lysates were included to calibrate the semiquantitative measurement. **(B)** Relative endogenous mRNA levels of p21, p53, and RB were measured in SiHa cells transfected with miR-375 inhibitor, -mimic, or NS control using qRT-PCR. **(C)** One hundred thousand SiHa cells were seeded on 24-well plate and the number of cells was counted by trypan blue exclusion staining assay 48 h post-transfection. **(D)** Protein levels of p21, p53, and RB in HeLa cells transfected with miR-375 inhibitor, -mimic, or NS control were measured by Western blot analysis. **(E)** Relative p21 mRNA levels were measured in HeLa cells transfected with miR-375 inhibitor, -mimic, or NS control. **(F)** Trypan blue exclusion staining assay was used to analyze the proliferation rate of HeLa cells 48 h post-transcfection. **(G)** Flow cytometry analysis demonstrates G1 arrest of SiHa cells 48 h after transfection with miR-375-mimic compared to miR-375 inhibitor or NS control. **(H)** Protein levels of CIP2A, p53, and p21 in SiHa cells transfected with si-CIP2A and/or si-p53 were measured by Western blot analysis. **(I)** Protein levels of CIP2A, p53, and p21 in SiHa cells transfected with si-p53 and/or miR-375-mimic were measured by Western blot analysis. **(J)** mRNA levels of p21 in SiHa cells transfected with si-p53 and/or miR-375-mimic were measured by qRT-PCR. The concentrations of siRNA or miRNA used in panels **H, I**, and **J** were 10 nM and 25 nM, respectively. Results are expressed as mean ± SD from three independent experiments. *p < 0.05, ***p < 0.001, and ****p < 0.0001.

HPV-positive cervical or oropharyngeal cancers contain wild type p53 and/or RB, and inactivation of these tumor suppressors are driven by E6 and E7 proteins produced from high-risk HPVs [28-30]. Cellular E6AP forms a complex with E6, inhibiting p53 by ubiquitin-dependent degradation [31]. E7 degrades RB, activating cell proliferation genes [32,33]. Therefore, SiHa and HeLa cells are appropriate representative model systems for studying HPV16 and HPV18, because they are known to contain wild type p53 and RB, respectively [28]. To examine the downstream effect of miR-375-mediated regulation of HPV transcripts and E6AP, we examined the expression of crucial tumor suppressors related to these pathways. In SiHa cells, miR-375-mimic transfection increased p53 and RB protein levels (Figure 3A). Interestingly, p21, which is a major downstream player in p53-mediated tumor suppression, showed dramatic protein level elevation after miR-375-mimic transfection (Figure 3A). p21 transcript levels significantly increased in cells with miR-375-mimic transfection (Figure 3B) and this increase could be explained by p53 protein level elevation (Figure 3A). The increase of p53 and RB proteins was not a result of transcriptional activation of corresponding genes, which indicates minimal changes in *de novo* proteins (Figure 3B). Therefore, it is more reasonable to interpret the increase as rescue of p53 and RB proteins by the miR-375-mediated suppression of E6 and E7 (Figure 1). A significant decrease in cell proliferation (~50%) was observed after miR-375-mimic transfection, compared to cells transfected with NS control (Figure 3C). These results showed that the rescue of tumor suppressors by miR-375-mediated regulation of E6 and E7 viral oncoproteins led to reduced proliferation of HPV-positive cancer cells.

Similar results were observed in HeLa cells. p21, p53, and RB protein levels dramatically increased and p21 mRNA levels significantly elevated in HeLa cells 48 h post-transfection of miR-375-mimic (Figure 3D and 3E). We observed about 40% reduction of cell proliferation after 48 h (Figure 3F). p21, p53, and RB are important cell cycle regulators [34-36]. Thus, flow cytometry was used to measure the cellular DNA content of cells transfected with miR-375-mimic or inhibitor to determine the cell cycle status. We observed a dramatic increase in G1-arrested cells and a reduced amount of S and G2/M phase cells 48 h post-transfection of miR-375-mimic, compared to NS control (~23%, p < 0.001, Figure 3G). Together, these results explain how miR-375 inhibits proliferation of these HPV-positive cancer cells.

### miR-375 control on CIP2A-MYC pathway also contributes to p21 elevation

In our previous study, we identified CIP2A as a target of miR-375 in oral cancer [3]. CIP2A protects MYC from dephosphorylation by PP2A and as a result prevents its proteolytic degradation [37], and MYC is a transcriptional repressor of p21 [38]. Thus, the potential involvement of miR-375 regulation of CIP2A-MYC in this pathway was investigated. We used siRNAs to knockdown either CIP2A or p53 to examine to effects of these proteins on p21 expression. Knockdown of p53 dramatically reduced p21 protein levels (76% reduction, Figure 3H). We also found a potential relevance between CIP2A and p53. Silencing CIP2A led to 33% increase of p53 (Figure 3H) and knockdown of p53 increased CIP2A to 40-80% (Figure 3H and 3I). While how p53 regulates CIP2A is unknown, it is clear that CIP2A plays a protective role for MYC [37], and that MYC impairs transactivation of p53 in human cancer cells [39-41]. Interestingly, knockdown of CIP2A increased p21 by 69% (Figure 3H), and increase of p21 by CIP2A silencing was also observed in p53-knockdown cells (a slight increase from 24% to 33%, Figure 3H). This result indicated that induction of p21 by miR-375 is mainly due to the elevation of its major transcriptional activator p53, and miR-375-mediated reduction of its transcriptional repressor MYC also contributes to p21 increase.

The effect of miR-375-mimic was examined in p53-knockdown SiHa cells in order to validate the contribution of CIP2A-MYC in miR-375-mediated increase in p21. Consistent with our previous observation [3], CIP2A was suppressed by miR-375 in SiHa cells (21% reduction, Figure 3I). Despite the variation in the increases of p53 and p21 shown in Figure 3A and 3I, the miR-375-mediated induction of p53 and p21 was reproducible in these separated experimental conditions. The reduced elevation shown in Figure 3I, compared to Figure 3A, may be affected by the co-transfection with si-GFP. Importantly, when miR-375-mimic was transfected into p53-knockdown cells, there was a dramatic increase of p21 (increase from 14% to 41%, Figure 3I). Since si-p53 efficiently silenced p53 proteins to a barely detectable level, miR-375-mediated increase of p21 is regulated by alternative pathways besides the major transactivator p53 (Figure 3I). Transcripts of p21 were significantly elevated by miR-375, as well as in p53-knockdown cells (Figure 3J). This indicates that in addition to p53-mediated activation of p21, miR-375 can activate p21 by regulating its suppressor MYC in the absence of p53.

### miR-375 increases p21 expression by inducing p53 and suppressing CIP2A-MYC

**Figure 4:**
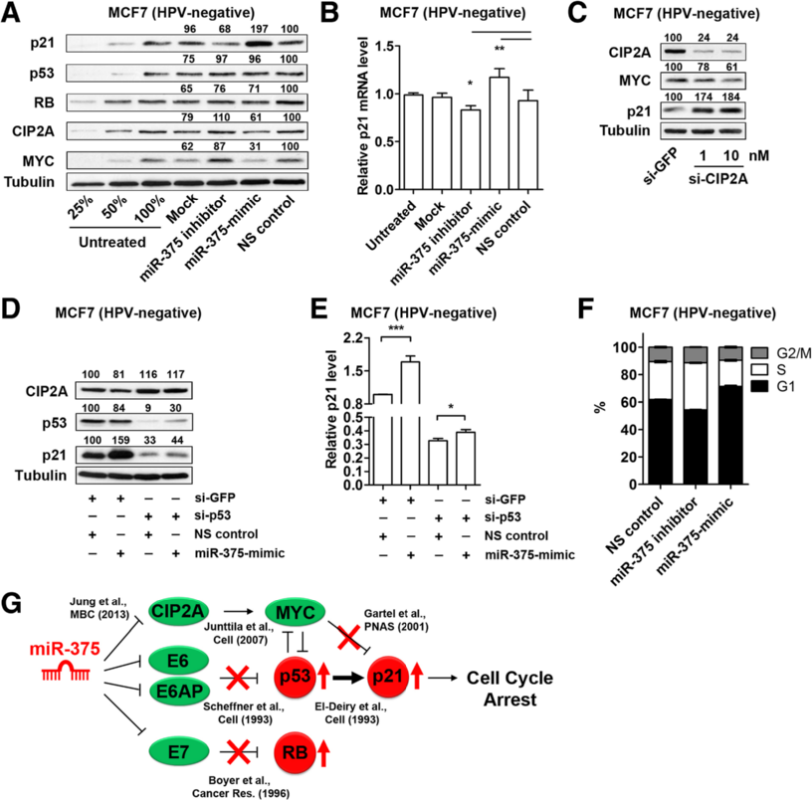
**miR-375 control on CIP2A-MYC pathway also contributes to p21 elevation. (A)** p21, p53, RB, CIP2A, and MYC protein levels in MCF7 cells transfected with miR-375 inhibitor, -mimic, or NS control were measured by Western blot analysis. Tubulin expression was used as internal control. 25%, 50%, 100% amounts of untreated cell lysates were included to calibrate the semiquantitative measurement. **(B)** Transfection with miR-375-mimic significantly upregulated p21 mRNA in MCF7. Relative endogenous p21 mRNA levels were measured in MCF7 cells transfected with miR-375 inhibitor, -mimic, or NS control for 48 h using qRT-PCR. **(C)** CIP2A and MYC protein levels were effectively silenced by si-CIP2A transfection with 1 and 10 nM concentrations for 48 h. Increased p21 protein levels were detected in si-CIP2A dose-dependent manner. 10 nM of si-GFP was used as a control. **(D)** Protein levels of CIP2A, p53, and p21 in MCF7 cells transfected with si-p53 and/or miR-375-mimic were measured by Western blot analysis. **(E)** mRNA levels of p21 in MCF7 cells transfected with si-p53 and/or miR-375-mimic were measured by qRT-PCR. **(F)** Flow cytometry analysis demonstrates G1 arrest of MCF7 cells 48 h after transfection with miR-375-mimic compared to miR-375 inhibitor or NS control. The concentrations of siRNA or miRNA used in panels **D, E**, and **F** were 10 nM and 25 nM, respectively. **(G)** Schematic depiction of miR-375-mediated repression of CIP2A, E6, E6AP, and E7 in HPV16-positive cells that simultaneously increases tumor suppressor p53, p21, and RB, and causes cell cycle arrest. Results are expressed as mean ± SD from three independent experiments. *p < 0.05, **p < 0.01, and ***p < 0.001.

We selected MCF7 as an HPV-negative model system to compare functional differences of miR-375 in HPV-positive versus HPV-negative cells. It is advantageous to use MCF7 cells because they are known to carry wild type p53 and RB [42,43]. This information allows us to rule out potential unexpected consequences that mutations in p53 and RB would generate. The same set of proteins that were examined in SiHa cells (p21, p53, and RB) were analyzed in MCF7 cells after transfecting miR-375 inhibitor, -mimic, or NS control (Figure 4A). Interestingly, transfection of miR-375-mimic did not change p53 and RB protein levels, compared to NS control transfected MCF7 cells. In contrast, p21 protein levels increased in miR-375-mimic transfected cells by 197% (Figure 4A). Despite the decrease of E6AP in miR-375-mimic transfected MCF7 cells (31% compared to NS control, data not shown), p53 protein level remained unaffected (Figure 4A). This finding confirms that miR-375-mediated suppression of E6 is required for p53 elevation. A significant increase of p21 transcripts was also observed in miR-375-mimic transfected MCF7 cells (120% compared to NS control, Figure 4B), although these fold changes were not as dramatic as those shown in HPV-positive cells (SiHa, ~330%, Figure 3B; HeLa, 350%, Figure 3E). MCF7 cells have relatively higher level of endogenous miR-375 (~27 Ct values by real-time PCR) than SiHa or HeLa cells. As a result, unlike no change observed in HPV-positive cells (Figure 3) with low miR-375 levels, transfection of miR-375 inhibitor in MCF7 cells decreased the level of p21 mRNA indicating that suppressing miR-375 led to repression of p21 (Figure 4B). Because p53 protein levels were not induced by miR-375-mimic transfection, an alternative explanation is required to explain the apparent increase of p21 transcriptional activation by miR-375 in MCF7 cells.

As described in our previous study [3], we confirmed that miR-375 suppressed CIP2A expression in MCF7 cells. About 40% and 70% reduction of CIP2A and MYC protein levels, respectively, were observed in miR-375-mimic transfected MCF7 cells, compared to NS control transfection (Figure 4A). This reduction indicates functional regulation of these molecules by miR-375 in this cell line. siRNA-mediated silencing of CIP2A causes degradation of MYC [3,37]. Using this strategy, CIP2A knockdown resulted in MYC reduction and p21 elevation in a siRNA dose-dependent manner (Figure 4C). Transfection of miR-375-mimic in p53-knockdown MCF7 cells increased the protein expression of p53 and p21 (Figure 4D) as well as p21 transcription (Figure 4E). The moderate increase of p53 shown in Figure 4D (compare lane 3 and 4, 9% versus 30%) indicated the potential of an HPV-independent pathway to activate p53. Since a miRNA can regulate multiple targets, this observation would require further investigation to fully understand how miR-375 induced p53 in HPV-negative cancer cells. Nevertheless, together with the results in Figure 3, these data suggested that replenishment of miR-375 can reactivate the expression of p21 through rescue of p53 from HPV E6, and miR-375-mediated suppression of CIP2A-MYC also contributes to the rescue of p21 in HPV-positive cancer cells. Although an increased percentage of G1 phase cells were observed in miR-375-mimic transfected cells compared to NS control (~10%, p < 0.001), this increase was not dramatic as that shown in SiHa cells (compare Figure 3G and 4F). Since MCF7 cells contained relatively higher level of miR-375 than SiHa cells, miR-375 inhibitor transfection showed reduction of G1 phase cells.

Collectively, miR-375 mediates co-regulation of CIP2A, E6, E6AP, and E7, and rescues the expression of major tumor suppressors p21, p53, and RB. Given the fact that p53 and MYC cross regulates each other [39-41,44], it is important to note that activation of p53 and suppression of MYC can be cooperated by miR-375, and results in activation of p21. As a consequence, activation of these tumor suppressors inhibit cell cycle progression and suppresses HPV-positive cancer cell proliferation (Figure 4G).

### miR-375-mediated repression of HPV16 E6, E6AP, and CIP2A activates p53-RB-p21 network and suppresses telomerase activity

**Figure 5:**
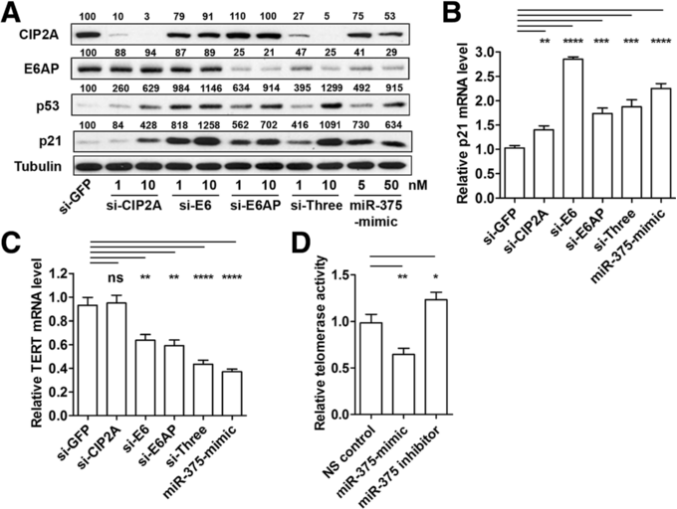
**miR-375-mediated repression of HPV16, E6AP, and CIP2A activates the p53-p21 network and suppresses telomerase activity. (A)** miR-375 demonstrates upregulation of p53 and p21 comparable to that of the single or combined CIP2A, E6, and E6AP knockdown. CIP2A, E6AP, p53, and p21 protein levels in SiHa cells transfected with siRNA targeting CIP2A, HPV16-E6, and E6AP (si-CIP2A, si-E6, and si-E6AP, respectively) were analyzed by Western blot. 1 nM and 10 nM siRNA concentrations and 5 nM and 50 nM for miR-375-mimic were used for transfection. si-Three is a combination of the three siRNAs indicated above. 10 nM of si-GFP was used as a control. Tubulin expression was used as internal control. **(B)** The increase in p21 protein levels correlate to its mRNA levels. Relative endogenous p21 mRNA levels transfected with siRNAs or miR-375 were measured using qRT-PCR. **(C)** miR-375 exerted a similar or stronger reduction in TERT mRNA levels when compared to E6 and E6AP knockdown in SiHa cells. **(D)** SiHa cells transfected with miR-375-mimic significantly reduced telomerase activity. Relative telomerase activities in SiHa cells transfected with NS control, miR-375-mimic, and miR-375 inhibitor were measured by SYBR real-time PCR TRAP assay. Heat-inactivated telomerase extracts were used to normalize this data. *p < 0.05, **p < 0.01, ***p < 0.001, and ****p < 0.0001. ns, not significant.

Because our data suggested that the absence of miR-375 in HPV-positive cancer cells leads to uncontrolled expression of miR-375 targets (HPV transcripts, E6AP, and CIP2A) and downstream molecules (p53, p21, RB, and MYC), we used siRNAs to silence individual miR-375 targets and examined their relative importance. Semiquantitative analyses of CIP2A, E6, and E6AP silencing by specific siRNA were conducted using two different siRNA concentrations (1 and 10 nM, Figure 5A). The effect of miR-375 was also examined using two different miR-375-mimic concentrations (5 and 50 nM) for parallel comparison. Due to the lack of an efficient antibody for E6 protein detection, its known target, p53, was quantitated as an E6 functional surrogate. Compared to si-GFP transfected cells, miR-375 transfection with two different siRNA concentrations confirmed the decrease of CIP2A and E6AP and the increase of p53 and p21 (Figure 5A). Individual siRNA transfection (si-CIP2A, si-E6, and si-E6AP) increased both p53 and p21 protein levels in concentration-dependent manners. Combination of three siRNAs (si-Three) showed an effect similar to using miR-375-mimic to reduce CIP2A and E6AP and elevate p53 and p21 (Figure 5A). Despite some technical difficulties in comparing the efficiency of each siRNA, silencing individual components of this pathway exclusively led to transcriptional activation of p21, as demonstrated in p21 mRNA analysis (Figure 5B). This data demonstrated that miR-375 increases p21 transcription possibly by coordinated regulation of CIP2A, HPV E6, and E6AP in this HPV-positive cancer cell.

Interestingly, proteins involved in the aforementioned pathways are well-known regulatory molecules for telomerase activity. MYC and E6 are transcriptional activators of TERT [45], while p53, p21, and RB are transcriptional repressors of TERT [46-49]. Therefore, we hypothesized that miR-375-mediated coordinated regulation of these components may lead to TERT transcriptional regulation. CIP2A knockdown alone was not sufficient to regulate TERT transcription, but silencing E6 or E6AP reduced TERT transcripts in SiHa cells (37% and 41% reduction, respectively, Figure 5C). Downregulation of the TERT transcript was most prominent when three siRNAs were used together compared to si-GFP control (57% reduction, p < 0.0001) and miR-375 exerted comparable or an even stronger reduction (~63% reduction, p < 0.0001, Figure 5C). The telomeric repeat amplification protocol assay incorporated with SYBR real-time PCR was used to measure telomerase activities in SiHa cells transfected with miR-375-mimic or inhibitor. About 35% reduced activity was observed in miR-375-mimic transfected cells whereas miR-375 inhibitor exhibited an opposite effect showing ~20% increase (Figure 5D). Taken together, this data shows miR-375 could regulate overall telomerase activity by reducing TERT transcription via CIP2A, E6, and/or E6AP in SiHa cells.

### miR-375 modulates TERT nuclear translocation by regulating 14-3-3ζ

**Figure 6:**
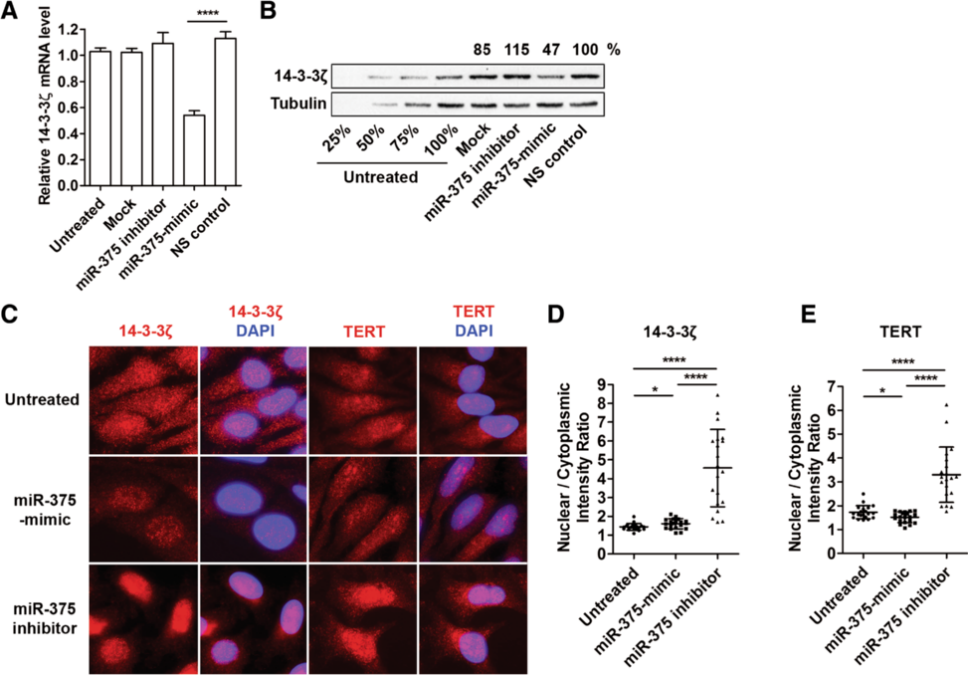
**miR-375 modulates TERT nuclear translocation by regulating 14-3-3ζ. (A)** Relative endogenous 14-3-3ζ mRNA levels were measured in HeLa cells transfected with miR-375 inhibitor, -mimic, or NS control using qRT-PCR. **(B)** In parallel, Western Blot analysis measured 14-3-3ζ protein level changes in cells transfected with miR-375 inhibitor, -mimic, or NS control. Tubulin expression was used as an internal control. 25%, 50%, 75%, 100% amounts of untreated cell lysates were included to calibrate the semiquantitative measurement. **(C)** Indirect immunofluorescence was used to monitor cellular localization of 14-3-3ζ and TERT 48 h post-transfection with miR-375-mimic or inhibitor in HeLa cells. Nuclei were counter-stained with DAPI. **(D)** Quantification of nuclear/cytoplasmic fluorescence intensities of 14-3-3ζ. **(E)** Quantification of nuclear/cytoplasmic fluorescence intensities of TERT. Data represents the mean ± SD for nuclear/cytoplasmic fluorescence ratio of 20 cells per image. *p < 0.05, ****p < 0.0001.

14-3-3 proteins interact with a wide variety of proteins involved in regulating many cellular processes [50]. 14-3-3 protein isoforms θ and ζ inhibit CRM1/exportin-1 binding of the TERT nuclear export signal motif. These isoforms share a conserved C-terminal domain that binds TERT and enhances TERT nuclear localization [51]. Interestingly, 14-3-3ζ has been identified as a miR-375 target [24]. We confirmed that miR-375-mimic represses 14-3-3ζ mRNA and protein levels ~50% in HeLa cells (Figure 6A and 6B). This data suggests the possibility that miR-375-mediated repression of 14-3-3ζ may affect the nuclear localization of TERT. 14-3-3ζ and TERT localization was examined by indirect immunofluorescence of HeLa cells transfected with miR-375-mimic or inhibitor. Overall fluorescence intensity for 14-3-3ζ was reduced in miR-375-mimic transfected cells compared to untreated cells and cells transfected with miR-375 inhibitors (Figure 6C). This result was expected as miR-375 inhibited *de novo* protein synthesis (Figure 6B). Comparing the ratio of 14-3-3ζ nuclear/cytoplasmic localization in untreated cells, miR-375-mimic transfected cells, and miR-375 inhibitor transfected cells, untreated and miR-375-mimic transfected cells had minimal differences (~10%) while miR-375 inhibitor treated cells demonstrated a dramatic increase (~300%) in this ratio (Figure 6D). This prominent decrease in cytoplasmic 14-3-3ζ expression could explain the slight increase of the nuclear/cytoplasmic ratio of 14-3-3ζ in miR-375-mimic transfected cells compared to untreated cells. Similarly, minimal changes in TERT nuclear localization were observed in miR-375-mimic transfected cells (12% decrease) and miR-375 inhibitor transfection enhanced nuclear localization of TERT (190% increase, Figure 6C and 6E). These results indicate that miR-375 modulates nuclear localization of 14-3-3ζ and TERT.

## Discussion

**Figure 7:**
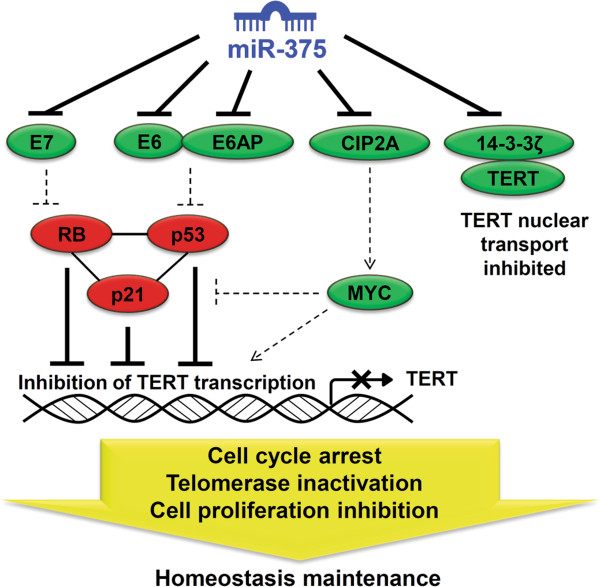
**Schematic model of miR-375 as a master regulator in HPV-positive cancers.** miR-375-mediated regulation simultaneously suppresses HPV E7, E6, E6AP, CIP2A, and 14-3-3ζ. As a result, the degradation of tumor suppressors p53 and RB is prevented, and p21 protein level increases. miR-375-mediated repression of CIP2A leads to degradation of oncogene MYC and also enhances p21 expression. Increase of transcriptional repressors (p53, p21, RB) and decrease of transcriptional activators (E6 and MYC) promotes TERT transcriptional reduction. miR-375-mediated repression of 14-3-3ζ inhibits nuclear translocation of TERT, thereby contributes to reduce telomerase activity. Overall, high miR-375 levels results in cell cycle arrest, telomerase inactivation and cell proliferation inhibition, which maintain homeostasis of normal epithelial cells. Solid lines represent “activated process” and dotted lines represent “inhibited process.” Molecules repressed or activated by miR-375 are distinguished by green and red colors, respectively.

Here we show the importance of miR-375 as a master suppressor of multiple oncogenic components involved in HPV-associated carcinogenesis. Repression mediated via a single miRNA has modest inhibitory effects on hundreds of targets (typically less than two-fold), but the strength of miRNA to change the gene-regulatory network comes from the cumulative reduction of multiple components of a pathway that enables miRNA to elicit biological changes [52-54]. Our results clearly show how miR-375 simultaneously coordinates regulation of HPV E6/E7, E6AP, and CIP2A to inhibit TERT transcription, and represses 14-3-3ζ to block TERT nuclear localization. Importantly, replenishment of miR-375 causes repression of these targets and rescues the major tumor suppressor network p53-p21-RB which is also involved in transcriptional repression of TERT. As a consequence, in the presence of miR-375, cell cycle arrest, telomerase inactivation, and proliferation inhibition maintain cellular homeostasis (Figure 7).

The loss of miR-375 is important for development of several types of cancers such as oral cancer [3], head and neck cancer [55-58], cervical cancer [59], hepatocellular cancer [60], oesophageal cancer [61], and gastric cancer [24,62]. Our study shows that low expression of miR-375 in HPV-associated cancers also contribute to carcinogenesis promoted by high-risk HPVs. Replenishment of miR-375 in HPV-positive cervical cancer cell lines and oropharyngeal cell line significantly reduces the levels of HPV transcripts. Moreover, miR-375 plays important role in regulating E6AP which is a protein required for HPV-mediated p53 degradation. It is important to note the oncogenic properties of high-risk HPVs. E6 and E7 can degrade important tumor suppressors p53, p21, and RB, and promote cellular machinery dysfunction required for homeostasis [33,63]. E6, in conjunction with cellular protein E6AP, targets p53 and leads to its proteosomal degradation [31]. p53 degradation inhibits p53-mediated apoptosis, reduces p21-mediated cell cycle regulation, and destabilizes chromosomes [64-66]. E7 oncoprotein binds and degrades RB via ubiquitin-mediated degradation [33]. RB degradation releases E2F, promoting transcriptional activation of many cell proliferation genes [32]. E7 protein also inactivates p21 and disrupts cell cycle control of normal human epithelial cells [67]. Therefore, our finding is that miR-375 critically suppresses HPV E6 and E7 expression, and as a result, maintains regular expressions of tumor suppressor genes in these epithelial cells.

HPV produces polycistronic mRNAs that contain E6, E7, and E1 regions [12] and alternative splicing on HPV polycistronic transcripts is important for E6 and E7 oncoprotein expressions [68]. Unspliced E6 coding region is essential for full-length E6 protein production. However, splicing on E6 region that prevents full-length E6 from being expressed and elevates E7 expression [68,69]. It has been suggested that alternative splicing of E6/E7 transcripts of high-risk HPVs occurs depending on epithelial growth factor (EGF). In the presence of EGF, equivalent amount of E6 and another isoform E6*I are produced, whereas E6*I expression becomes dominant in EGF depleted condition. Splicing events may occur during differentiation where EGF and EGF receptor levels change during differentiation of suprabasal epithelial cells [70]. While it is clear that alternative splicing of HPV transcripts have functional relevance on biological processes such as cell cycle and differentiation, miR-375-mediated regulation of HPV transcripts are not affected by alternative splicing because the putative miR-375 binding sites are located either on E7 or E1 region and not on E6 region. Since the primers and probes used for the real time qPCR analyses of E6 or E7 transcript are located in regions that are common in all spliced transcripts, our data support that miR-375-mediated regulation of HPV transcripts will be independent of E6 mRNA splicing as long as the transcripts have downstream E7 and E1 regions still in place.

Our work on miR-375 adds to the growing body of evidence implicating the association between miRNA and HPV in cancers. Previous studies have shown the influence of HPV genes on miRNA expressions. For example, HPV16 E6 modulates miR-218 levels [71] and E6-mediated degradation p53 leads to suppression of miR-34a [72], miR-23b [73], and miR-145 [74]. E7 protein downregulates miR-203 upon differentiation of suprabasal epithelial cells [75]. These observations are not surprising because E6 and E7 modulate numbers of major transcription factors such as MYC, p53, and E2F which are upstream molecules for a large number of these miRNA genes. MYC induces expression of miR-17-92 family, and represses a number of miRNAs including miR-30 family [76]. p53 not only transcriptionally activates miR-34a [77] but also regulates biogenesis of mature miR-15a, miR-16-1, miR-23a, miR-26a, miR-103, miR-143, miR-145, miR-203, and miR-206 [78]. p53 also function as transcriptional repressor mediated by E2F to modulate miRNA expressions [79]. Previous studies suggest a potential of miRNAs involved in HPV regulation by showing that high levels of miR-203 in differentiating suprabasal cells have inhibitory effect on HPV amplification [75] and miR-125b interaction with late gene of HPV6 [80]. These reports indicate a high possibility for other miRNA-mediated regulation for HPV transcripts but experimental validation is still needed [81]. Therefore, our finding that a single cellular miRNA regulates HPV transcripts suggests a need for a systemic investigation to unveil cellular miRNA-mediated direct and/or indirect regulations of HPV genes. Our cell culture system used HPV-positive cancer cell lines that express endogenous HPV viral genes, and this system has limitation to mimic the natural HPV infections in epithelial cells since HPVs do not replicate in cell culture. Therefore, additional investigations are required to understand whether sufficient endogenous miR-375 levels in epithelial cells can suppress HPV viral gene expressions by using organotypic (raft) tissue culture system that mimic fully differentiated epithelium [82,83].

## Conclusions

Our study showed mechanistically for the first time how cellular miRNA interacts with high-risk HPV transcripts and reduces their expression. Present data demonstrated that supplement of miR-375 in HPV-positive cancer cells coordinates the regulation of HPV E6/E7, E6AP, CIP2A, and 14-3-3ζ, which secures the expression of tumor suppressors p53, RB, and p21, and regulates telomerase activity. Therefore, loss of miR-375 during HPV-mediated carcinogenesis is likely to lead mis-regulation of this network. Our current finding suggests a need of development of therapeutic approaches to increase miR-375 levels in HPV-associated cancers, which will suppress oncoproteins but in turn re-express major tumor suppressors and ultimately lead to improve clinical outcomes.

## Materials and methods

### Patient specimens, cell culture, and transfection

Three oropharyngeal tumors were obtained from the Tissue Bank at the Moffitt Cancer Center (protocol no. MCC-15370) and approved by the Institutional Review Board of the University of South Florida (no. 106444). SiHa, HEK293, and HeLa cells were cultured as described in previous publication [84], and MCF7 cells were cultured in DMEM supplemented with 10% fetal bovine serum, 100 μg/mL streptomycin, and 100 U/mL penicillin. UPCI:SCC090 is an HPV16-positive tongue cancer cell line provided from Dr. Susanne Gollin at the University of Pittsburgh [85]. Transfection was performed using Lipofectamine 2000. Sequences of siRNAs were designed according to previous reports: CIP2A, 5′- CUGUGGUUGUGUUUGCACUdTdT-3′ [37]; HPV16 E6, 5′-ACCGTTGTGTGATTTGTTAdTdT-3′ [86]; E6AP, 5′-CAACTCCTGCTCTGAGATAdTdT-3′ [86]. siRNAs were synthesized from Thermo Fisher Scientific (Waltham, MA). p53 siRNA was purchased from Thermo Fisher Scientific (D003329-05-0002). GFP siRNA (Thermo Fisher Scientific, D-001300-01-20) was used as a control.

### Western blot and antibodies

Western blotting was performed and analyzed as described [84]. Primary antibodies used were mouse anti-MYC (9E-10, Santa Cruz Biotechnology), mouse monoclonal anti-CIP2A (2G10, Novus Biologicals), rabbit anti-E6AP (H-182, Santa Cruz Biotechnology), mouse anti-p53 (DO-1, Santa Cruz Biotechnology), rabbit anti-RB1 (10048-2-Ig, Proteintech), rabbit anti-p21 (10355-1-AP, Proteintech), rabbit anti-14-3-3ζ (C-16, Santa Cruz Biotechnology), and mouse anti-tubulin (Sigma-Aldrich).

### Dual luciferase assay and site-directed mutagenesis

The luciferase assay was performed in HEK293 cells using the dual luciferase reporter assay system (Promega) and analyzed as described [3]. cDNA fragments of HPV16 transcript and E6AP 3′UTR were subcloned into a pMiR-Target vector between *EcoR*I and *Not*I restriction enzyme sites downstream of Firefly luciferase (FL) coding sequence (OriGene Technologies, Rockville, MD). Four nucleotides in the seed binding sites were mutated to disrupt the miRNA-mRNA interaction as described previously [3]. Renilla luciferase reporter was used to normalize the data. All primers used for cloning luciferase construction and mutagenesis are listed in Additional file 1: Table S1.

### Cell cycle analysis by flow cytometry

SiHa and MCF7 cells were harvested 48 h post-transfection and cellular DNA content was measured using Cycletest Plus DNA Reagent Kit (BD Biosciences, San Jose, CA). Samples were analyzed on a FACS Calibur flow cytometer (BD Biosciences) and the data was collected using Cell Quest Software, Version 3.3 (BD Biosciences). Flow cytometry measured the fluorescence from propidium iodide as an indication of DNA content during cell cycle. Fluorescence was excited by a 15 mW laser emitting at 488 nm. Emission was collected using a 585 nm +/- 21 nm bandpass filter. Data for 2×10^4^ events were collected per sample. Cell cycle analysis was performed using Modfit software, version 3.1 (Verity Software House, Topsham, ME).

### Real-time quantitative telomeric repeat amplification protocol (RQ-TRAP) assay and TaqMan qRT-PCR

TaqMan qRT-PCR was performed as described [3,87]. Customized primers and probes for detection of E6 and E7 from HPV16 or HPV18 were selected from a published report [88]: HPV16 E6-F, 5′- GCACCAAAAGAGAACTGCAATGTT-3′; HPV16 E6-R, 5′-AGTCATATACCTCACGTCGCAGTA-3′; HPV16 E6-probe, 5′-GGACCCACAGGAGCGACCCAGAAAGTTA-3′; HPV16 E7-F, 5′-CAAGTGTGACTCTACGCTTCGG-3′; HPV16 E7-R, 5′-GTGGCCCATTAACAGGTCTTCCAA-3′; HPV16 E7-probe, 5′-TGCGTACAAAGCACACACGTAGACATTCGT-3′; HPV18 E6-F, 5′-CTATAGAGGCCAGTGCCATTCG-3′; HPV18 E6-R, 5′-TTATACTTGTGTTTCTCTGCGTCG-3′; HPV18 E6-probe, 5′-CAACCGAGCACGACAGGAACGACTCCA-3′; HPV18 E7-F, 5′-TAATCATCAACATTTACCAGCCCG-3′; HPV18 E7-R, 5′-CGTCTGCTGAGCTTTCTACTACTA-3′; HPV18 E7-probe, 5′-CGAGCCGAACCACAACGTCACACAATGTT-3′. Semi-quantitative PCR detected full length HPV16 transcripts of E6, E7, and E6/E7/E1. HPV16 E6-F1, 5′-ATGCACCAAAAGAGAACTGC-3′; HPV16 E6-R1, 5′-TTACAGCTGGGTTTCTCTACG-3′; HPV16 E7-F1, 5′-ATGCATGGAGATACACCTACA-3′; HPV16 E7-R1, 5′-TTATGGTTTCTGACAACAGATGG-3′. E6/E7/E1 gene product was detected using HPV16 E6-F1 and E1 reverse cloning primer described in Additional file 1: Table S1. E6 isoforms were clearly detected in the tumors and cell lines in our study (Additional file 1: Figure S2). Telomerase activity was detected using TRAPeze Telomerase Detection Kit (Millipore) combined with SYBR green real-time PCR [89]. Briefly, cells were resuspended in CHAPS lysis buffer, incubated on ice for 30 min and obtained the supernatant after centrifuging at 12,000 g for 20 min at 4°C. Heat-inactivated samples were incubated at 85°C for 10 min and were used to normalize the total telomerase activity. SYBR green real-time and TaqMan qRT-PCR were performed using the Applied Biosystems StepOne Real-Time PCR machine.

### Indirect immunofluorescence and quantification of fluorescence

Indirect immunofluorescence was performed as described [3,90,91] using rabbit anti-14-3-3ζ and rabbit anti-TERT. Alexa 568 goat anti-rabbit Ig were used as secondary antibodies. Images were taken at ×400 magnification using Zeiss Axiovert 200 M microscope. Fluorescence intensities in the nuclear and cytoplasmic regions were quantified using ImageJ (http://rsbweb.nih.gov/ij/). Nuclear/Cytoplasmic ratios were calculated from the mean of fluorescence intensities measured in a defined circular area within the nucleus or cytoplasm of each cell. Twenty cells were captured and ratios calculated for quantification.

### Statistical analysis

Experiments were conducted at least trice, and data are shown as mean ± SD. Statistical analyses for miR-375-mimic transfected samples were performed by comparing the values of NS control transfected samples unless indicated in figures. Statistical analyses were performed using two-tailed Student’s *t* test using GraphPad Prism 4.0 (Graph Pad Software, La Jolla, CA).

### Consent

Written informed consent was obtained from patients for the publication of this report.

## Abbreviations

(miRNA): microRNA; (HPV): Human papillomavirus; (E6AP): E6-associated protein; (RB): Retinoblastoma protein 1; (TERT): Telomerase reverse transcriptase; (nt): Nucleotide; (UTR): 3′ untranslated region; (FL): Firefly luciferase; (CIP2A): Cancerous Inhibitor of PP2A.

## Competing interests

The authors declare that they have no competing interests.

## Authors’ contributions

HMJ and EKLC designed research and analyzed data. HMJ and BLP carried out experiments. HMJ and EKLC wrote the paper. All authors have read and approved the final manuscript.

## Supplementary Material

Additional file 1: Figure S1 Mutation of single miR-375 binding site does not abolish the repression effect. **Figure S2.** PCR amplification of E6, E7, and E6E7E1 transcripts in SiHa, SCC090, and three HPV16 positive oropharyngeal tumors (T1-T3). **Table S1.** Primer sequences for cloning and mutagenesis. Click here for file

## References

[B1] Bartel DP MicroRNAs: target recognition and regulatory functions Cell 2009 136 215 233 10.1016/j.cell.2009.01.002 19167326 PMC3794896

[B2] Reczko M Maragkakis M Alexiou P Grosse I Hatzigeorgiou AG Functional microRNA targets in protein coding sequences Bioinformatics 2012 28 771 776 10.1093/bioinformatics/bts043 22285563

[B3] Jung HM Patel RS Phillips BL Wang H Cohen DM Reinhold WC Chang LJ Yang LJ Chan EK Tumor suppressor miR-375 regulates MYC expression via repression of CIP2A coding sequence through multiple miRNA-mRNA interactions Mol Biol Cell 2013 24 1638 1648 10.1091/mbc.E12-12-0891 23552692 PMC3667718

[B4] Friedman RC Farh KK Burge CB Bartel DP Most mammalian mRNAs are conserved targets of microRNAs Genome Res 2009 19 92 105 18955434 10.1101/gr.082701.108 PMC2612969

[B5] zur Hausen H de Villiers EM Human papillomaviruses Annu Rev Microbiol 1994 48 427 447 10.1146/annurev.mi.48.100194.002235 7826013

[B6] Gillison ML Chaturvedi AK Lowy DR HPV prophylactic vaccines and the potential prevention of noncervical cancers in both men and women Cancer 2008 113 3036 3046 10.1002/cncr.23764 18980286 PMC6264789

[B7] Jemal A Simard EP Dorell C Noone AM Markowitz LE Kohler B Eheman C Saraiya M Bandi P Saslow D Cronin KA Watson M Schiffman M Henley SJ Schymura MJ Anderson RN Yankey D Edwards BK Annual Report to the Nation on the Status of Cancer, 1975-2009, featuring the burden and trends in human papillomavirus (HPV)-associated cancers and HPV vaccination coverage levels J Natl Cancer Inst 2013 105 175 201 10.1093/jnci/djs491 23297039 PMC3565628

[B8] Chang YE Laimins LA Microarray analysis identifies interferon-inducible genes and Stat-1 as major transcriptional targets of human papillomavirus type 31 J Virol 2000 74 4174 4182 10.1128/JVI.74.9.4174-4182.2000 10756030 PMC111932

[B9] Nees M Geoghegan JM Hyman T Frank S Miller L Woodworth CD Papillomavirus type 16 oncogenes downregulate expression of interferon-responsive genes and upregulate proliferation-associated and NF-kappaB-responsive genes in cervical keratinocytes J Virol 2001 75 4283 4296 10.1128/JVI.75.9.4283-4296.2001 11287578 PMC114174

[B10] Barnard P McMillan NA The human papillomavirus E7 oncoprotein abrogates signaling mediated by interferon-alpha Virology 1999 259 305 313 10.1006/viro.1999.9771 10388655

[B11] Ronco LV Karpova AY Vidal M Howley PM Human papillomavirus 16 E6 oncoprotein binds to interferon regulatory factor-3 and inhibits its transcriptional activity Genes Dev 1998 12 2061 2072 10.1101/gad.12.13.2061 9649509 PMC316980

[B12] Yugawa T Kiyono T Molecular mechanisms of cervical carcinogenesis by high-risk human papillomaviruses: novel functions of E6 and E7 oncoproteins Rev Med Virol 2009 19 97 113 10.1002/rmv.605 19156753

[B13] Walboomers JM Jacobs MV Manos MM Bosch FX Kummer JA Shah KV Snijders PJ Peto J Meijer CJ Munoz N Human papillomavirus is a necessary cause of invasive cervical cancer worldwide J Pathol 1999 189 12 19 10.1002/(SICI)1096-9896(199909)189:1<12::AID-PATH431>3.0.CO;2-F 10451482

[B14] Syrjanen S The role of human papillomavirus infection in head and neck cancers Ann Oncol 2010 21 Suppl 7 vii243 vii245 20943622 10.1093/annonc/mdq454

[B15] Munger K Howley PM Human papillomavirus immortalization and transformation functions Virus Res 2002 89 213 228 10.1016/S0168-1702(02)00190-9 12445661

[B16] Blackburn EH Switching and signaling at the telomere Cell 2001 106 661 673 10.1016/S0092-8674(01)00492-5 11572773

[B17] Moyzis RK Buckingham JM Cram LS Dani M Deaven LL Jones MD Meyne J Ratliff RL Wu JR A highly conserved repetitive DNA sequence, (TTAGGG) n, present at the telomeres of human chromosomes Proc Natl Acad Sci U S A 1988 85 6622 6626 10.1073/pnas.85.18.6622 3413114 PMC282029

[B18] Harley CB Futcher AB Greider CW Telomeres shorten during ageing of human fibroblasts Nature 1990 345 458 460 10.1038/345458a0 2342578

[B19] Vaziri H Schachter F Uchida I Wei L Zhu X Effros R Cohen D Harley CB Loss of telomeric DNA during aging of normal and trisomy 21 human lymphocytes Am J Hum Genet 1993 52 661 667 8460632 PMC1682068

[B20] Hemann MT Strong MA Hao LY Greider CW The shortest telomere, not average telomere length, is critical for cell viability and chromosome stability Cell 2001 107 67 77 10.1016/S0092-8674(01)00504-9 11595186

[B21] Kim NW Piatyszek MA Prowse KR Harley CB West MD Ho PL Coviello GM Wright WE Weinrich SL Shay JW Specific association of human telomerase activity with immortal cells and cancer Science 1994 266 2011 2015 10.1126/science.7605428 7605428

[B22] Klingelhutz AJ Foster SA McDougall JK Telomerase activation by the E6 gene product of human papillomavirus type 16 Nature 1996 380 79 82 10.1038/380079a0 8598912

[B23] Granger MP Wright WE Shay JW Telomerase in cancer and aging Crit Rev Oncol Hematol 2002 41 29 40 10.1016/S1040-8428(01)00188-3 11796230

[B24] Tsukamoto Y Nakada C Noguchi T Tanigawa M Nguyen LT Uchida T Hijiya N Matsuura K Fujioka T Seto M Moriyama M MicroRNA-375 is downregulated in gastric carcinomas and regulates cell survival by targeting PDK1 and 14-3-3zeta Cancer Res 2010 70 2339 2349 10.1158/0008-5472.CAN-09-2777 20215506

[B25] Miranda KC Huynh T Tay Y Ang YS Tam WL Thomson AM Lim B Rigoutsos I A pattern-based method for the identification of MicroRNA binding sites and their corresponding heteroduplexes Cell 2006 126 1203 1217 10.1016/j.cell.2006.07.031 16990141

[B26] del Moral-Hernandez O Lopez-Urrutia E Bonilla-Moreno R Martinez-Salazar M Arechaga-Ocampo E Berumen J Villegas-Sepulveda N The HPV-16 E7 oncoprotein is expressed mainly from the unspliced E6/E7 transcript in cervical carcinoma C33-A cells Arch Virol 2010 155 1959 1970 10.1007/s00705-010-0787-9 20865289

[B27] Lewis BP Burge CB Bartel DP Conserved seed pairing, often flanked by adenosines, indicates that thousands of human genes are microRNA targets Cell 2005 120 15 20 10.1016/j.cell.2004.12.035 15652477

[B28] Scheffner M Munger K Byrne JC Howley PM The state of the p53 and retinoblastoma genes in human cervical carcinoma cell lines Proc Natl Acad Sci U S A 1991 88 5523 5527 10.1073/pnas.88.13.5523 1648218 PMC51909

[B29] Crook T Wrede D Tidy J Scholefield J Crawford L Vousden KH Status of c-myc, p53 and retinoblastoma genes in human papillomavirus positive and negative squamous cell carcinomas of the anus Oncogene 1991 6 1251 1257 1650445

[B30] Dai M Clifford GM le Calvez F Castellsague X Snijders PJ Pawlita M Herrero R Hainaut P Franceschi S Human papillomavirus type 16 and TP53 mutation in oral cancer: matched analysis of the IARC multicenter study Cancer Res 2004 64 468 471 10.1158/0008-5472.CAN-03-3284 14744758

[B31] Scheffner M Huibregtse JM Vierstra RD Howley PM The HPV-16 E6 and E6-AP complex functions as a ubiquitin-protein ligase in the ubiquitination of p53 Cell 1993 75 495 505 10.1016/0092-8674(93)90384-3 8221889

[B32] Iaquinta PJ Lees JA Life and death decisions by the E2F transcription factors Curr Opin Cell Biol 2007 19 649 657 10.1016/j.ceb.2007.10.006 18032011 PMC2268988

[B33] Boyer SN Wazer DE Band V E7 protein of human papilloma virus-16 induces degradation of retinoblastoma protein through the ubiquitin-proteasome pathway Cancer Res 1996 56 4620 4624 8840974

[B34] Sherr CJ McCormick F The RB and p53 pathways in cancer Cancer Cell 2002 2 103 112 10.1016/S1535-6108(02)00102-2 12204530

[B35] Du W Searle JS The rb pathway and cancer therapeutics Curr Drug Targets 2009 10 581 589 10.2174/138945009788680392 19601762 PMC3151466

[B36] Niculescu AB 3rd Chen X Smeets M Hengst L Prives C Reed SI Effects of p21 (Cip1/Waf1) at both the G1/S and the G2/M cell cycle transitions: pRb is a critical determinant in blocking DNA replication and in preventing endoreduplication Mol Cell Biol 1998 18 629 643 9418909 10.1128/mcb.18.1.629 PMC121530

[B37] Junttila MR Puustinen P Niemela M Ahola R Arnold H Bottzauw T Ala-aho R Nielsen C Ivaska J Taya Y Lu SL Lin S Chan EK Wang XJ Grenman R Kast J Kallunki T Sears R Kahari VM Westermarck J CIP2A inhibits PP2A in human malignancies Cell 2007 130 51 62 10.1016/j.cell.2007.04.044 17632056

[B38] Gartel AL Ye X Goufman E Shianov P Hay N Najmabadi F Tyner AL Myc represses the p21 (WAF1/CIP1) promoter and interacts with Sp1/Sp3 Proc Natl Acad Sci U S A 2001 98 4510 4515 10.1073/pnas.081074898 11274368 PMC31865

[B39] Ceballos E Munoz-Alonso MJ Berwanger B Acosta JC Hernandez R Krause M Hartmann O Eilers M Leon J Inhibitory effect of c-Myc on p53-induced apoptosis in leukemia cells. Microarray analysis reveals defective induction of p53 target genes and upregulation of chaperone genes Oncogene 2005 24 4559 4571 10.1038/sj.onc.1208652 15856024

[B40] Eischen CM Weber JD Roussel MF Sherr CJ Cleveland JL Disruption of the ARF-Mdm2-p53 tumor suppressor pathway in Myc-induced lymphomagenesis Genes Dev 1999 13 2658 2669 10.1101/gad.13.20.2658 10541552 PMC317106

[B41] Ceballos E Delgado MD Gutierrez P Richard C Muller D Eilers M Ehinger M Gullberg U Leon J c-Myc antagonizes the effect of p53 on apoptosis and p21WAF1 transactivation in K562 leukemia cells Oncogene 2000 19 2194 2204 10.1038/sj.onc.1203541 10822369

[B42] Lee EY To H Shew JY Bookstein R Scully P Lee WH Inactivation of the retinoblastoma susceptibility gene in human breast cancers Science 1988 241 218 221 10.1126/science.3388033 3388033

[B43] Wosikowski K Regis JT Robey RW Alvarez M Buters JT Gudas JM Bates SE Normal p53 status and function despite the development of drug resistance in human breast cancer cells Cell Growth Differ 1995 6 1395 1403 8562478

[B44] Aguda BD Kim Y Kim HS Friedman A Fine HA Qualitative network modeling of the Myc-p53 control system of cell proliferation and differentiation Biophys J 2011 101 2082 2091 10.1016/j.bpj.2011.09.052 22067145 PMC3207173

[B45] Wu KJ Grandori C Amacker M Simon-Vermot N Polack A Lingner J Dalla-Favera R Direct activation of TERT transcription by c-MYC Nat Genet 1999 21 220 224 10.1038/6010 9988278

[B46] Kusumoto M Ogawa T Mizumoto K Ueno H Niiyama H Sato N Nakamura M Tanaka M Adenovirus-mediated p53 gene transduction inhibits telomerase activity independent of its effects on cell cycle arrest and apoptosis in human pancreatic cancer cells Clin Cancer Res 1999 5 2140 2147 10473098

[B47] Kanaya T Kyo S Hamada K Takakura M Kitagawa Y Harada H Inoue M Adenoviral expression of p53 represses telomerase activity through down-regulation of human telomerase reverse transcriptase transcription Clin Cancer Res 2000 6 1239 1247 10778946

[B48] Shats I Milyavsky M Tang X Stambolsky P Erez N Brosh R Kogan I Braunstein I Tzukerman M Ginsberg D Rotter V p53-dependent down-regulation of telomerase is mediated by p21waf1 J Biol Chem 2004 279 50976 50985 10.1074/jbc.M402502200 15371422

[B49] Crowe DL Nguyen DC Rb and E2F-1 regulate telomerase activity in human cancer cells Biochim Biophys Acta 2001 1518 1 6 10.1016/S0167-4781(00)00296-7 11267653

[B50] Gardino AK Smerdon SJ Yaffe MB Structural determinants of 14-3-3 binding specificities and regulation of subcellular localization of 14-3-3-ligand complexes: a comparison of the X-ray crystal structures of all human 14-3-3 isoforms Semin Cancer Biol 2006 16 173 182 10.1016/j.semcancer.2006.03.007 16678437

[B51] Seimiya H Sawada H Muramatsu Y Shimizu M Ohko K Yamane K Tsuruo T Involvement of 14-3-3 proteins in nuclear localization of telomerase EMBO J 2000 19 2652 2661 10.1093/emboj/19.11.2652 10835362 PMC212742

[B52] Small EM Olson EN Pervasive roles of microRNAs in cardiovascular biology Nature 2011 469 336 342 10.1038/nature09783 21248840 PMC3073349

[B53] Selbach M Schwanhausser B Thierfelder N Fang Z Khanin R Rajewsky N Widespread changes in protein synthesis induced by microRNAs Nature 2008 455 58 63 10.1038/nature07228 18668040

[B54] Lim LP Lau NC Garrett-Engele P Grimson A Schelter JM Castle J Bartel DP Linsley PS Johnson JM Microarray analysis shows that some microRNAs downregulate large numbers of target mRNAs Nature 2005 433 769 773 10.1038/nature03315 15685193

[B55] Nohata N Hanazawa T Kikkawa N Mutallip M Sakurai D Fujimura L Kawakami K Chiyomaru T Yoshino H Enokida H Nakagawa M Okamoto Y Seki N Tumor suppressive microRNA-375 regulates oncogene AEG-1/MTDH in head and neck squamous cell carcinoma (HNSCC) J Hum Genet 2011 56 595 601 10.1038/jhg.2011.66 21753766

[B56] Avissar M Christensen BC Kelsey KT Marsit CJ MicroRNA expression ratio is predictive of head and neck squamous cell carcinoma Clin Cancer Res 2009 15 2850 2855 10.1158/1078-0432.CCR-08-3131 19351747 PMC2669849

[B57] Harris T Jimenez L Kawachi N Fan JB Chen J Belbin T Ramnauth A Loudig O Keller CE Smith R Prystowsky MB Schlecht NF Segall JE Childs G Low-level expression of miR-375 correlates with poor outcome and metastasis while altering the invasive properties of head and neck squamous cell carcinomas Am J Pathol 2012 180 917 928 10.1016/j.ajpath.2011.12.004 22234174 PMC3349885

[B58] Hui AB Bruce JP Alajez NM Shi W Yue S Perez-Ordonez B Xu W O'Sullivan B Waldron J Cummings B Gullane P Siu L Liu FF Significance of dysregulated metadherin and microRNA-375 in head and neck cancer Clin Cancer Res 2011 17 7539 7550 10.1158/1078-0432.CCR-11-2102 22031094

[B59] Wang F Li Y Zhou J Xu J Peng C Ye F Shen Y Lu W Wan X Xie X miR-375 is down-regulated in squamous cervical cancer and inhibits cell migration and invasion via targeting transcription factor SP1 Am J Pathol 2011 179 2580 2588 10.1016/j.ajpath.2011.07.037 21945323 PMC3204087

[B60] Ladeiro Y Couchy G Balabaud C Bioulac-Sage P Pelletier L Rebouissou S Zucman-Rossi J MicroRNA profiling in hepatocellular tumors is associated with clinical features and oncogene/tumor suppressor gene mutations Hepatology 2008 47 1955 1963 10.1002/hep.22256 18433021

[B61] Kong KL Kwong DL Chan TH Law SY Chen L Li Y Qin YR Guan XY MicroRNA-375 inhibits tumour growth and metastasis in oesophageal squamous cell carcinoma through repressing insulin-like growth factor 1 receptor Gut 2012 61 33 42 10.1136/gutjnl-2011-300178 21813472

[B62] Ding L Xu Y Zhang W Deng Y Si M Du Y Yao H Liu X Ke Y Si J Zhou T MiR-375 frequently downregulated in gastric cancer inhibits cell proliferation by targeting JAK2 Cell Res 2010 20 784 793 10.1038/cr.2010.79 20548334

[B63] Scheffner M Werness BA Huibregtse JM Levine AJ Howley PM The E6 oncoprotein encoded by human papillomavirus types 16 and 18 promotes the degradation of p53 Cell 1990 63 1129 1136 10.1016/0092-8674(90)90409-8 2175676

[B64] el-Deiry WS Tokino T Velculescu VE Levy DB Parsons R Trent JM Lin D Mercer WE Kinzler KW Vogelstein B WAF1, a potential mediator of p53 tumor suppression Cell 1993 75 817 825 10.1016/0092-8674(93)90500-P 8242752

[B65] Kessis TD Slebos RJ Nelson WG Kastan MB Plunkett BS Han SM Lorincz AT Hedrick L Cho KR Human papillomavirus 16 E6 expression disrupts the p53-mediated cellular response to DNA damage Proc Natl Acad Sci U S A 1993 90 3988 3992 10.1073/pnas.90.9.3988 8387205 PMC46431

[B66] White AE Livanos EM Tlsty TD Differential disruption of genomic integrity and cell cycle regulation in normal human fibroblasts by the HPV oncoproteins Genes Dev 1994 8 666 677 10.1101/gad.8.6.666 7926757

[B67] Helt AM Funk JO Galloway DA Inactivation of both the retinoblastoma tumor suppressor and p21 by the human papillomavirus type 16 E7 oncoprotein is necessary to inhibit cell cycle arrest in human epithelial cells J Virol 2002 76 10559 10568 10.1128/JVI.76.20.10559-10568.2002 12239337 PMC136576

[B68] Zheng ZM Tao M Yamanegi K Bodaghi S Xiao W Splicing of a cap-proximal human Papillomavirus 16 E6E7 intron promotes E7 expression, but can be restrained by distance of the intron from its RNA 5’ cap J Mol Biol 2004 337 1091 1108 10.1016/j.jmb.2004.02.023 15046980

[B69] Tang S Tao M McCoy JP Jr Zheng ZM The E7 oncoprotein is translated from spliced E6*I transcripts in high-risk human papillomavirus type 16- or type 18-positive cervical cancer cell lines via translation reinitiation J Virol 2006 80 4249 4263 10.1128/JVI.80.9.4249-4263.2006 16611884 PMC1472016

[B70] Rosenberger S De-Castro Arce J Langbein L Steenbergen RD Rosl F Alternative splicing of human papillomavirus type-16 E6/E6* early mRNA is coupled to EGF signaling via Erk1/2 activation Proc Natl Acad Sci U S A 2010 107 7006 7011 10.1073/pnas.1002620107 20351270 PMC2872467

[B71] Martinez I Gardiner AS Board KF Monzon FA Edwards RP Khan SA Human papillomavirus type 16 reduces the expression of microRNA-218 in cervical carcinoma cells Oncogene 2008 27 2575 2582 10.1038/sj.onc.1210919 17998940 PMC2447163

[B72] Wang X Wang HK McCoy JP Banerjee NS Rader JS Broker TR Meyers C Chow LT Zheng ZM Oncogenic HPV infection interrupts the expression of tumor-suppressive miR-34a through viral oncoprotein E6 RNA 2009 15 637 647 10.1261/rna.1442309 19258450 PMC2661824

[B73] Au Yeung CL Tsang TY Yau PL Kwok TT Human papillomavirus type 16 E6 induces cervical cancer cell migration through the p53/microRNA-23b/urokinase-type plasminogen activator pathway Oncogene 2011 30 2401 2410 10.1038/onc.2010.613 21242962

[B74] Shi M Du L Liu D Qian L Hu M Yu M Yang Z Zhao M Chen C Guo L Wang L Song L Ma Y Guo N Glucocorticoid regulation of a novel HPV-E6-p53-miR-145 pathway modulates invasion and therapy resistance of cervical cancer cells J Pathol 2012 228 148 157 10.1002/path.3997 22287315

[B75] Melar-New M Laimins LA Human papillomaviruses modulate expression of microRNA 203 upon epithelial differentiation to control levels of p63 proteins J Virol 2010 84 5212 5221 10.1128/JVI.00078-10 20219920 PMC2863797

[B76] Chang TC Yu D Lee YS Wentzel EA Arking DE West KM Dang CV Thomas-Tikhonenko A Mendell JT Widespread microRNA repression by Myc contributes to tumorigenesis Nat Genet 2008 40 43 50 10.1038/ng.2007.30 18066065 PMC2628762

[B77] Chang TC Wentzel EA Kent OA Ramachandran K Mullendore M Lee KH Feldmann G Yamakuchi M Ferlito M Lowenstein CJ Arking DE Beer MA Maitra A Mendell JT Transactivation of miR-34a by p53 broadly influences gene expression and promotes apoptosis Mol Cell 2007 26 745 752 10.1016/j.molcel.2007.05.010 17540599 PMC1939978

[B78] Suzuki HI Yamagata K Sugimoto K Iwamoto T Kato S Miyazono K Modulation of microRNA processing by p53 Nature 2009 460 529 533 10.1038/nature08199 19626115

[B79] Brosh R Shalgi R Liran A Landan G Korotayev K Nguyen GH Enerly E Johnsen H Buganim Y Solomon H Goldstein I Madar S Goldfinger N Borresen-Dale AL Ginsberg D Harris CC Pilpel Y Oren M Rotter V p53-Repressed miRNAs are involved with E2F in a feed-forward loop promoting proliferation Mol Syst Biol 2008 4 229 19034270 10.1038/msb.2008.65 PMC2600669

[B80] Nuovo GJ Wu X Volinia S Yan F di Leva G Chin N Nicol AF Jiang J Otterson G Schmittgen TD Croce C Strong inverse correlation between microRNA-125b and human papillomavirus DNA in productive infection Diagn Mol Pathol 2010 19 135 143 10.1097/PDM.0b013e3181c4daaa 20736742 PMC4284817

[B81] Zheng ZM Wang X Regulation of cellular miRNA expression by human papillomaviruses Biochim Biophys Acta 1809 2011 668 677 10.1016/j.bbagrm.2011.05.005 PMC3175324 21616186

[B82] Meyers C Frattini MG Hudson JB Laimins LA Biosynthesis of human papillomavirus from a continuous cell line upon epithelial differentiation Science 1992 257 971 973 10.1126/science.1323879 1323879

[B83] McCance DJ Kopan R Fuchs E Laimins LA Human papillomavirus type 16 alters human epithelial cell differentiation in vitro Proc Natl Acad Sci U S A 1988 85 7169 7173 10.1073/pnas.85.19.7169 2459699 PMC282145

[B84] Jung HM Phillips BL Patel RS Cohen DM Jakymiw A Kong WW Cheng JQ Chan EK Keratinization-associated miR-7 and miR-21 Regulate Tumor Suppressor Reversion-inducing Cysteine-rich Protein with Kazal Motifs (RECK) in Oral Cancer J Biol Chem 2012 287 29261 29272 10.1074/jbc.M112.366518 22761427 PMC3436145

[B85] Ragin CC Reshmi SC Gollin SM Mapping and analysis of HPV16 integration sites in a head and neck cancer cell line Int J Cancer 2004 110 701 709 10.1002/ijc.20193 15146560

[B86] Kelley ML Keiger KE Lee CJ Huibregtse JM The global transcriptional effects of the human papillomavirus E6 protein in cervical carcinoma cell lines are mediated by the E6AP ubiquitin ligase J Virol 2005 79 3737 3747 10.1128/JVI.79.6.3737-3747.2005 15731267 PMC1075713

[B87] Jakymiw A Patel RS Deming N Bhattacharyya I Shah P Lamont RJ Stewart CM Cohen DM Chan EK Overexpression of dicer as a result of reduced let-7 MicroRNA levels contributes to increased cell proliferation of oral cancer cells Genes Chromosomes Cancer 2010 49 549 559 10.1002/gcc.20765 20232482 PMC2859695

[B88] Cattani P Siddu A D’Onghia S Marchetti S Santangelo R Vellone VG Zannoni GF Fadda G RNA (E6 and E7) assays versus DNA (E6 and E7) assays for risk evaluation for women infected with human papillomavirus J Clin Microbiol 2009 47 2136 2141 10.1128/JCM.01733-08 19403762 PMC2708475

[B89] Wege H Chui MS Le HT Tran JM Zern MA SYBR Green real-time telomeric repeat amplification protocol for the rapid quantification of telomerase activity Nucleic Acids Res 2003 31 E3 E13 12527792 10.1093/nar/gng003 PMC140528

[B90] Soo Hoo L Zhang JY Chan EKL Cloning and characterization of a novel 90 kDa ‘companion’ auto-antigen of p62 overexpressed in cancer Oncogene 2002 21 5006 5015 10.1038/sj.onc.1205625 12118381

[B91] Katz J Jakymiw A Ducksworth MK Stewart CM Bhattacharyya I Cha S Chan EK CIP2A expression and localization in oral carcinoma and dysplasia Cancer Biol Ther 2010 10 694 699 10.4161/cbt.10.7.12895 21068540 PMC3230513

