# Supplementary material for: miR-375 activates p21 and suppresses telomerase activity by coordinately regulating HPV E6/E7, E6AP, CIP2A, and 14-3-3ζ
Source: Mol Cancer. 2014 Apr 8;13:80. doi: 10.1186/1476-4598-13-80 (PMC4021670; doi:10.1186/1476-4598-13-80)
Supplement: Additional file 1: Figure S1 — Mutation of single miR-375 binding site does not abolish the repression effect. Figure S2. PCR amplification of E6, E7, and E6E7E1 transcripts in SiHa, SCC090, and three HPV16 positive oropharyngeal tumors (T1-T3). Table S1. Primer sequences for cloning and mutagenesis. [file 1476-4598-13-80-S1.pdf]

miR-375 activates p21 and suppresses telomerase activity by coordinately regulating HPV E6/E7, E6AP, CIP2A, and 14-3-3ζ

Hyun Min Jung[1], Brittany L. Phillips[1], Edward K. L. Chan[1]

[1]Department of Oral Biology, University of Florida, 1395 Center Drive, Gainesville, FL 32610, USA

Additional File

Figure S1-2

Table S1

Figure S1

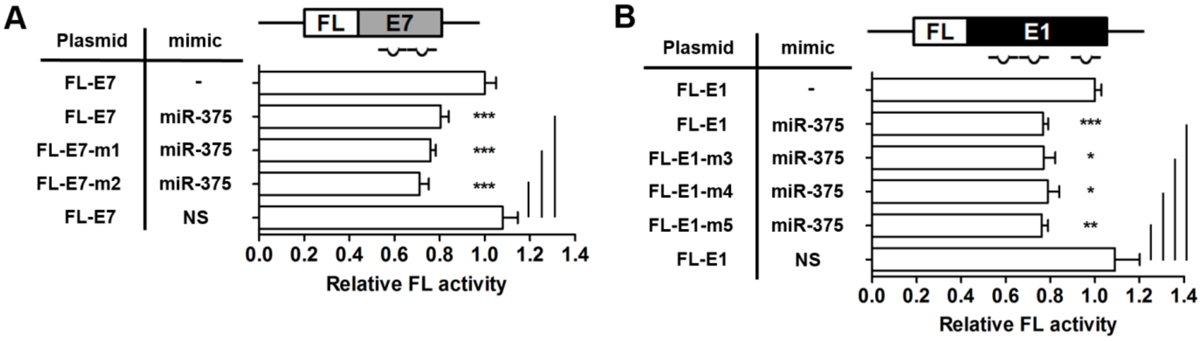

Figure S1. Mutation of single miR-375 binding site does not abolish the repression effect. (A) miR-375 interaction with E7 region was determined by relative FL expression from cells transfected with FL-E7 with or without miR-375-mimic transfection. Two individual mutations for putative miR-375 binding sites were generated (FL-E7-m1 or FL-E7-m2). (B) miR-375 interaction with E1 region was similarly determined. Three individual mutations were generated for each miR-375 binding site (FL-E1-m3, FL-E1-m4, and FL-E1-m5).

Figure S2

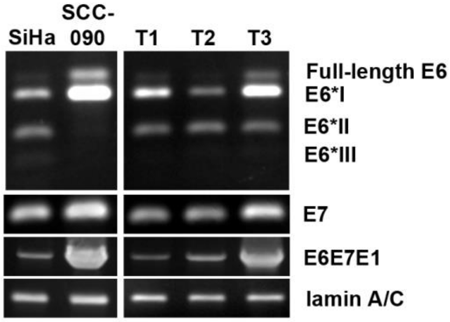

Figure S2. PCR amplification of E6, E7, and E6E7E1 transcripts in SiHa, SCC090, and three HPV16 positive oropharyngeal tumors (T1-T3). Lamin A/C was measured for internal control.

Table S1. Primer sequences for cloning and mutagenesis

Primer name	Sequences
HPV16-E7-F (for cloning)	5’-ggaattccatgcatgcagatacacctaca-3’
HPV16-E7-R (for cloning)	5’-gatcgcggccgcttaggtttctgagaacagatgg-3’
HPV16-E7-m1-F (for mutagenesis)	5'-aatagatggtccagctggacattgtgaaccggacagagcccattac-3'
HPV16-E7-m1-R (for mutagenesis)	5'-gtaatgggctctgtccggttcacaatgtccagctggaccatctatt-3'
HPV16-E7-m2-F (for mutagenesis)	5'-gtccagctggacaagcagaacccgtgtgagcccattacaatattgtaac-3'
HPV16-E7-m2-R (for mutagenesis)	5'-gttacaatattgtaatgggctcacacgggttctgcttgtccagctggac-3'
HPV16-E7-m1-2-F (for mutagenesis)	5'-ggttacaatattgtaatgggctcacaccggttcaccatgtccagctggaccatctatttc-3'
HPV16-E7-m1-2-R (for mutagenesis)	5'-gaaatagatggtccagctggacatggtgaaccggtgtgagcccattacaatattgtaacc-3'
HPV16-E1-F (for cloning)	5’-ctaggaattcaatgggcctacgataatga-3’
HPV16-E1-R (for cloning)	5’-gatcgcggccgctcataatgtgttagtattttgtcc-3’
HPV16-E1-m3-F (for mutagenesis)	5'-tgtgcaacaatgtgtagacattataaacgagcagaaaaaaatgttatgagtatgagtcaatggataaaata-3'
HPV16-E1-m3-R (for mutagenesis)	5'-tattttatccattgactcatactcataacatttttttctgctcgtttataatgtctacacattgttgcaca-3'
HPV16-E1-m4-F (for mutagenesis)	5'-cagaaaaaaaacaaatgagtatgagtcaatcgttttaatatagatgtgatagggtagatgatggag-3'
HPV16-E1-m4-R (for mutagenesis)	5'-ctccatcatctaccctatcacatctatattaaaacgattgactcatactcatttgttttttttctg-3'
HPV16-E1-m5-F (for mutagenesis)	5'-attaagtttgcacgaggacgaggtgttggaaaacgatggagactctttgc-3'
HPV16-E1-m5-R (for mutagenesis)	5'-gcaaagagtctccatcgttttccaacacctcgtcctcgtgcaaacttaat-3'
